# Tincture of Chloride of Iron

**Published:** 1891-09

**Authors:** Thomas J. Giblin


### TINCTURE OF CHLORIDE OF IRON.[1]

BY THOMAS J. GIBLIN, D.M.D.

The subject of my paper was suggested by the condition of the teeth so frequently noticed in our patients after the continued use of the tincture of chloride of iron as a medicine.

You will all, doubtless, recall having brought the teeth of a patient into proper condition by the careful use of the wonderful means of restoration at your command, and to have seen, after a comparatively short period of time, the same patient return and, to your surprise, exhibit in his teeth the ravages of decay. Upon inquiry, you learn that the patient, acting upon the advice of some physician, has been using the tincture of chloride of iron. The patient will usually inform you that he has taken for some time, through a tube, a solution of tincture of iron in water. This experience has been mine so frequently, of late, that I have judged the question of the cause of this trouble of sufficient importance to discuss it in my paper to-night, and in it rather to suggest an investigation than to propose a remedy.

What is the agent of which my paper treats? I will recall to your minds some facts concerning it. It is a mixture of alcohol and the solution of the chloride of iron, which has been made by pouring dilute hydrochloric acid upon a quantity of fine iron wire cut into small pieces, allowing it to stand during effervescence, after which it is boiled and filtered. To the filtered liquid is added hydro-

---

[1] Read before the Harvard Odontological Society, Thursday evening, March 26, 1891.

chloric acid. This mixture is poured slowly into nitric acid. After effervescence ceases, heat is applied until the disappearance of the nitrous odor. Dilute hydrochloric acid is added to make the whole equal 100.

The properties of the tincture of chloride of iron are thus described: It is a bright-brownish liquid of a slightly ethereal odor, a very astringent, styptic taste, and an acid reaction. It is decomposed by the alkalies, alkaline earths, and their carbonates, astringent vegetable infusions and mucilage of gum arabic. It is one of the most active and certain preparations of iron, and is much employed as a tonic. Its purpose is to supply the necessary integral portion of the red blood-corpuscles.

This is the preparation of whose effect, as witnessed in the dental chair, I desire to treat. That this tincture, in solution, does exert such deleterious effect upon the teeth is probably agreed to by all practising dentists. If there be an exception, he can be readily convinced by a simple experiment. Let him leave a tooth in a solution of one drachm of tincture of chloride of iron and one ounce of water for twenty-four hours. He will find the enamel softened and easily scraped.

Having admitted such an effect, what should be our course as dentists? There is a class of medical practitioners who especially endeavor to make little of dental medicine, and who usually think that the end justifies the means. Such will be satisfied to know that by the use of the tincture of chloride of iron they have supplied to the blood that element which will increase the activity of its functions, even though the result be disastrous to another important organ of the body. We should each call to the attention of the physicians of our acquaintance the clinical experiences we have had with the uses of this drug. It is not for us, as dentists, to tell them what medicine they shall use, or how they should prescribe it, but if they come into our field with their results we surely are privileged to criticise the means which produce such evils to the teeth.

We have a precedent in our own society to warrant this course. At our last meeting a prominent and scientific member of the medical profession called our attention to the possibility of our work producing results that might demand his care. Here we have the certain damage done by their medicine, which requires our work to repair. We appreciate and cordially receive his advice; I am sure we were all influenced by it. Why should not our words be equally well received? I feel sure they would, and that our medical friends would endeavor to prevent such action of this drug as we complain of.

That this is probable is shown by the experience of the past. When the effect of this medicine upon the teeth was formerly called to their attention, they prescribed it to be dissolved in water, and the solution to be taken through a tube. They believed that the injury was done by the free acid in the tincture, and that the solution in water would weaken its action, and, still further, that the use of a tube would prevent it altogether. These physicians were honest in their course, but mistaken. It is easily shown that water especially increases the destructive power of the tincture of iron on the lime salts of the teeth. If water be added to a neutral solution of the chloride of iron, it will decompose it, giving basic salts of iron and free hydrochloric acid. Certainly water is worse than useless in connection with the tincture of iron. When a strong solution of this tincture is used, it first forms a dense and hard oxide of iron, which closely adheres to the surface of the tooth, and serves it as a coat of mail, protecting it from the action of the free acid. The oxide formed in the water solution is very different in its nature, being flocculent and non-protecting to the teeth. As for the use of tubes, I believe that they have been shown to afford very little defence, perhaps because of almost universal carelessness or ignorance in their use.

I have said that I would not suggest a remedy, because I believe it not to be within the bounds of our professional field. We are not expected to trace, through all the organs of the body, the probable action and effect of any method of administering this drug or any other. If we suggest an investigation of the cause of evil results, we have fulfilled our duty, and have gone as far as professional etiquette permits, unless we are invited to give a further opinion. Our duty, in a humanitarian spirit, is to prevent the occurrence of decay in the teeth of our patients, as well as to repair the damage done.

We are, as a profession, justly proud of the progress we have made towards the consummation of our hopes of inhibiting bacteria, the exciting cause of decay.

I offer my paper to-night, trusting that some attention may be drawn to what I will term a secondary cause of decay.

